# Prediction of lymph node metastasis in lung adenocarcinoma using a PET/CT radiomics-based ensemble learning model and its pathological basis

**DOI:** 10.3389/fonc.2025.1618494

**Published:** 2025-08-25

**Authors:** Shulin Li, Fang Chen, Lei Wang, Zhiming Xiang

**Affiliations:** ^1^ Postgraduate Cultivation Base of Guangzhou University of Chinese Medicine, Panyu Central Hospital, Guangzhou, China; ^2^ Department of Radiology, The Affiliated Panyu Central Hospital, Guangzhou Medical University, Guangzhou, China; ^3^ Department of Pathology, The Affiliated Panyu Central Hospital, Guangzhou Medical University, Guangzhou, China

**Keywords:** lung adenocarcinoma, lymph node metastasis, positron emission tomography, radiomics, pathomics, stacking ensemble learning

## Abstract

**Objectives:**

Lymph node metastasis (LNM) is an important factor affecting the stage and prognosis of patients with lung adenocarcinoma. The purpose of this study is to explore the predictive value of the stacking ensemble learning model based on ^18^F-FDG PET/CT radiomic features and clinical risk factors for LNM in lung adenocarcinoma, and elucidate the biological basis of predictive features through pathological analysis.

**Methods:**

Ninety patients diagnosed with lung adenocarcinoma who underwent PET/CT were retrospectively analyzed and randomly divided into the training and testing sets in a 7:3 ratio. Stacking ensemble learning models were developed based on radiomic features combined with clinical risk factors. The predictive performance of each model was assessed through area under the curve (AUC). Additionally, Spearman’s correlation analysis was employed to investigate the association between features predicting LNM and pathological features.

**Results:**

Multifactorial logistic regression identified the bronchial cut-off sign and serum carcinoembryonic antigen (CEA) as clinical risk factors. The Stacking-combined model demonstrated superior diagnostic efficacy compared with logistic regression, random forest, and naive Bayes-combined models, with AUC values of 0.971 and 0.901 in the training and testing sets, respectively. Despite the absence of FDR-significant radiomic-pathomic correlations (all *q* > 0.05), exploratory analysis revealed nominal associations (uncorrected *P* < 0.05) for partial feature pairs. Crucially, radiomic features demonstrated strong associations with Ki-67 expression: PET_GLRLM_LongRunHigh GreyLevelEmphasis (r = 0.610, *q* < 0.001) and CT_INTENSITY-BASED_Intensity BasedEnergy (r = 0.332, *q* = 0.004).

**Conclusions:**

The stacking ensemble learning model based on ^18^F-FDG PET/CT radiomics demonstrates potential for predicting LNM in lung adenocarcinoma, and the quantitative analysis of radiomic features holds significant biological significance.

## 1 Introduction

Lung adenocarcinoma is the most predominant pathologic subtype of lung cancer, accounting for approximately 40% of all lung cancers (1, 2). Lymph node metastasis (LNM) is an important factor in patient survival and greatly influences patients’ staging and treatment approaches. The ninth edition of TNM classification emphasizes significant differences in 5-year survival rates based on the involvement of LNM, with 83%, 58%, 51%, 40%, and 28% for pN0, pN1, pN2a, pN2b, and pN3, respectively (3); and this gap is equally applicable to clinical staging. According to the latest guidelines of the National Comprehensive Cancer Network (NCCN), patients with no LNM (N0) or localized LNM (N1-2) typically undergo surgical resection. Non-surgical treatments are usually recommended for N3 patients (4, 5). Accurate assessment of LNM can provide a more adequate basis for optimizing clinical management strategies.

Pathological biopsy is the most reliable method for LNM in lung cancer; however, it is an invasive examination that may cause injury to patients, such as bleeding, infection, and pneumothorax. Additionally, it is challenging to sample some lymph nodes due to the special anatomical structure around them. In comparison, as a non-invasive and reproducible method, imaging is the most prevalent technique for evaluating N staging. Previous studies have demonstrated that Positron Emission Tomography-Computed Tomography (PET/CT) exhibits superior accuracy in assessing N staging compared to CT and Magnetic Resonance Imaging (MRI) (6–8). However, it is difficult to distinguish between benign and malignant lymph nodes with increased metabolism due to reactive hyperplasia, inflammation, granulomatous disease, and other lesions (9). With the rapid development of artificial intelligence technology, radiomics extracts features from medical images (CT, MRI, and PET) in a high-throughput manner (10). This approach extensively explores and analyzes image data to quantitatively assess the overall tumor heterogeneity (11, 12). For the past few years, radiomics based on ^18^F-FDG PET/CT has shown significant potential in predicting LNM in non-small cell lung cancer (NSCLC), and is anticipated to guide the selection of treatment strategies (13–19).

While radiomic features possess the potential to reflect tumor heterogeneity, their specific biological significance and clinical application value require further elucidation. Pathology directly reflects tumor information by analyzing microscopic tissue structures and cellular characteristics, providing a comprehensive biological context and a clinical validation basis for interpreting radiomic features. Among the histological subtypes of lung adenocarcinoma, micropapillary and solid subtypes tend to be more aggressive and have a strong association with LNM (20, 21). Nevertheless, conventional pathological diagnosis relies on subjective visual assessments, posing significant challenges to achieving uniformity and precision among different physicians. To overcome this limitation, pathomics, as an emerging interdisciplinary field that integrates pathology and omics techniques, provides a powerful tool for the in-depth analysis of pathological characteristics and histological subtypes of tumors (22–24). Combining pathomics information with radiomic features aids in clarifying the biological significance of image textures, thereby enhancing our understanding of features. There exists some evidence suggesting a cross-scale correlation between the two in various diseases (25–27). Therefore, a thorough investigation of the pathological features of lung adenocarcinoma can provide a more specific biological interpretation of radiomic features and improve the comprehension of tumor heterogeneity.

In summary, this study aims to develop a stacking ensemble learning model for predicting LNM in lung adenocarcinoma based on ^18^F-FDG PET/CT radiomics and attempts to elucidate the histomorphological basis of predictive features from a pathological perspective. This approach can deepen our insight into the role of radiomics as a “virtual biopsy”, thereby fostering its application and advancement in the field of precision medicine.

## 2 Materials and methods

### 2.1 Study design

**Figure 1:**
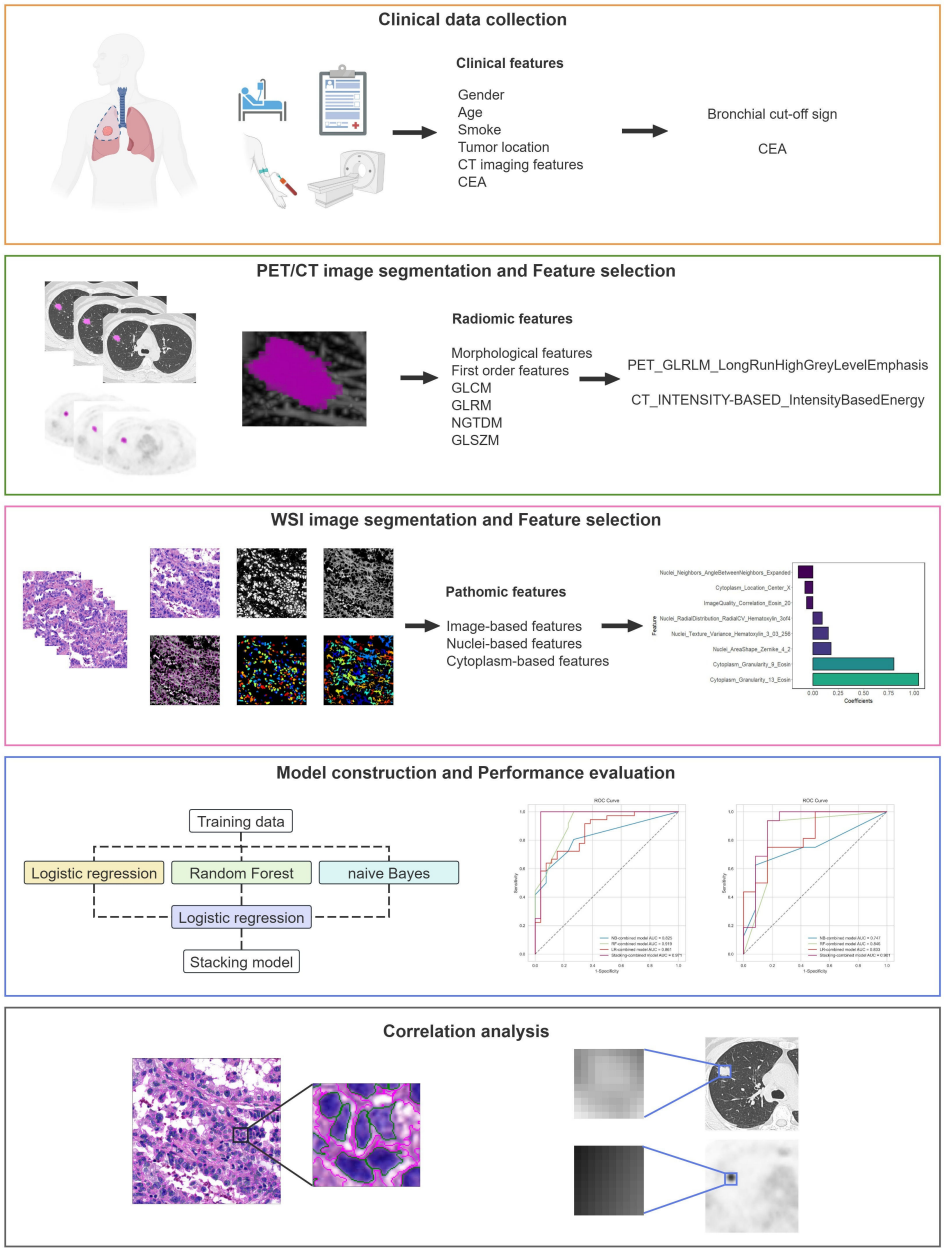
Flowchart of this study.

The study adhered to the CLEAR checklist for conducting and reporting experimental research, detailed in **Supplementary Data Sheet S1**. The flowchart of this study is shown in **Figure 1**, including clinical data collection, image acquisition, region of interest (ROI) segmentation, feature extraction and selection, model construction and performance evaluation, as well as correlation analysis.

### 2.2 Patients

A retrospective analysis was conducted on patients with lung adenocarcinoma who underwent a pretreatment ^18^F-FDG PET/CT examination at our hospital between May 2022 and April 2024. Inclusion criteria: (1) All patients used the identical ^18^F-FDG PET/CT equipment under uniform scanning conditions; (2) Lung adenocarcinoma was pathologically diagnosed for the first time; (3) Complete clinical and imaging records. Exclusion criteria: (1) Pure ground-glass nodule (pGGN) without FDG metabolism; (2) Indistinct tumor boundaries on ^18^F-FDG PET/CT images hindering sketch completion; (3) Previous tumor history or concurrent malignant neoplasms; (4) Chemotherapy, radiotherapy, targeted therapy, immunotherapy, and other anti-tumor treatments before ^18^F-FDG PET/CT examination. All selected patients were randomly divided into training and testing sets at a ratio of 7:3.

Clinical information, comprising gender, age, smoking history, semantic features (tumor location, lobulation sign, spiculation sign, pleural indentation sign, and bronchial cut-off sign), serum carcinoembryonic antigen (CEA) levels, and Ki-67 expression level was collected from medical records. The CEA levels were measured by electrochemiluminescence, with a reference range of 0 to 5 ng/ml. Hematoxylin/eosin (H&E) stained whole slide images (WSI) were acquired from some of these patients for further analysis. Criteria for metastatic lymph nodes: The gold standard for diagnosis is the pathological results. For suspicious lesions for which surgical/puncture pathology results could not be obtained, the final diagnosis relied on multiple examinations and subsequent follow-up images over a period of more than 3 months.

This study received approval from the Medical Ethics Committee of our hospital, thereby exempting patients from informed consent.

### 2.3 PET/CT image acquisition

All ^18^F-FDG PET/CT scans were performed on the same device (GE Discovery MI PET/CT, GE HealthCare, Waukesha, WI). All patients fasted for more than 6 hours before the imaging and had blood glucose levels below 11.1 mmol/L. After receiving an injection of 0.11-0.14 mCi/kg of ^18^F-FDG, patients rested quietly for approximately 60 minutes. A breath-holding CT scan was performed from the vertex of the skull to the mid thighs, and used for attenuation correction purposes as well as anatomic location of ^18^F-FDG uptake. The CT images were acquired using 64-slice helical CT with the following settings: 120 or 140 kV, automatic tube current technique, reconstructed layer thickness of 1.25 mm, a rotation time of 0.6 s, a pitch of 1.375, a matrix of 512×512, and lung window (window width, 1500 HU; window position, -700 HU). Subsequently, the PET images were acquired with a matrix of 128×128 and a layer thickness of 2.78 mm. Following data acquisition, attenuation correction and reconstruction procedures were conducted to generate PET, CT, and PET/CT fusion images for each three-dimensional scanning level (transverse, coronal, and sagittal planes), as well as the whole-body MIP maps of PET images.

### 2.4 PET/CT image processing and feature extraction

In this study, the lesion’s ROI delineation and feature extraction of PET/CT images were completed on LIFEx 7.4.0. The ROI was delineated layer by layer on the CT and PET images by two senior attending nuclear medicine physicians. The ROIs of PET images were semi-automatically delineated using a threshold of 42% of the SUVmax as the optimization criterion, and then resampled to 1mm×1mm×1mm (x, y, z) to standardize the voxel spacing. The feature extraction parameters were set to the default values. In the CT and PET ROIs, 179 radiomic features were extracted from the original images respectively, including 50 morphological features, 73 first-order statistics features, and 56 second-order feature parameters.

### 2.5 Radiomic feature selection

For missing data, the extracted PET and CT radiomic features were imputed with the median value and standardized by Z-score normalization. Subsequently, features exhibiting statistical differences were identified using the t-test or Mann-Whitney U test. Spearman correlation analysis was employed to remove features with high correlation, specifically those with a Spearman’s correlation coefficient greater than 0.9. The Max-Relevance Min-Redundancy (mRMR) method and Gradient Boosting Decision Tree (GBDT) algorithm were utilized to further diminish data dimensionality and isolate the most informative radiomic features.

### 2.6 WSI preparation and immunohistochemical analysis

To ensure an accurate assessment, tumors were re-evaluated by two pathologists with over five years of experience in diagnosing lung cancer. Each tumor was categorized in accordance with the WHO classification system for lung cancer (5th version), and the percentage of each histological component was recorded in 5% increments, determining the presence or absence of micropapillary/solid components in the lesions. Decisions were made through collaborative consultation and discussion in case of disagreement. A representative section of each patient was selected and digitized using Pathology Medical Image Analysis System IBL500 at a magnification of 40x, subsequently exporting the images in.svs format.

A mouse anti-human Ki-67 monoclonal antibody was used for the immunohistochemical detection. Positive and negative controls were set up separately, and cells with brownish-yellow nuclei were considered as positive cells. The number of Ki-67 positive tumor cells was counted under 400x microscope in five fields. The percentage of Ki-67 expression level positive staining of tumor cells in each field = the number of positive tumor cells in each field/total tumor cells in each field × 100%. The Ki-67 indices of five visual fields were calculated and averaged.

### 2.7 WSI processing and feature extraction

Tumor regions within WSIs were manually delineated utilizing Qupath 0.5.1 and the feature extraction were completed on CellProfiler 4.2.7 (29). To reduce the computational time, the delineated WSIs were segmented into patches with a field of view of 1024×1024 pixels. For each patient, 20 patches were randomly selected and were clear and unobstructed. All patches underwent color normalization utilizing the Vahadane method (28) for subsequent processing. An automated image processing workflow was developed utilizing CellProfiler to extract quantitative features based on images, tumor nuclei, and tumor cytoplasm with 225, 279, and 271, respectively. The Image-based features comprehensively evaluated each patch, including overall image quality, intensity, granularity, texture, and correlation between stained images. The Nuclei- and Cytoplasm-based features encompass a variety of characteristics, including the number of measured objects, their location, shape, intensity, granularity, texture, and spatial relationships. The mean value of each feature was calculated and aggregated to the WSI level for further analysis.

### 2.8 Pathomic feature selection

Initially, the extracted pathomic features were standardized by Z-score normalization for preprocessing. Spearman correlation analysis was employed to eliminate redundant information; if the correlation coefficient was greater than 0.9, one of them was retained. The final feature selection was performed using the least absolute shrinkage and selection operator (LASSO) algorithm with five-fold cross-validation. Features with non-zero coefficients were retained for correlation analysis.

### 2.9 Model construction

Clinical risk factors predictive of LNM in lung adenocarcinoma were identified by univariate and multivariate logistic regression analysis. Based on the stacking ensemble learning algorithm, a clinical model, a PET/CT radiomics model, and a combined model were developed. The stacking ensemble learning algorithm employed logistic regression (LR), random forest (RF), and naive Bayes (NB) as the base learners, with logistic regression (LR) serving as the meta-learner. Optimal model parameters were automatically determined using five-fold cross-validation, which shuffles data into 5 subsets, trains on 4, and validates on 1, repeating to minimize bias. Furthermore, three conventional machine learning algorithms—LR, RF, and NB—were utilized to develop individual combined models, which were compared to the Stacking-combined model.

### 2.10 Statistical analysis

The study was statistically analyzed using SPSS 26.0, R 4.4.1, and Python 3.9.1. Continuous variables were compared using the t-test or the Mann-Whitney U test. Categorical variables were compared using the Chi-square test or Fisher’s exact test.

Radiomic feature selection employed mRMR (scikit-learn 1.0.2, Python) and GBDT-based importance ranking (LightGBM 3.3.2, Python), whereas pathomic feature selection utilized LASSO regression (glmnet 4.1-8, R). Predictive models were constructed using scikit-learn 1.0.2 (Python) and evaluated with pROC 1.18.0 (R), with performance quantified by the area under the receiver operating characteristic curve (AUC). AUC differences were evaluated using the DeLong test, and decision curve analysis (DCA) was employed to evaluate the clinical utility of each model. Visualizations were generated with matplotlib 3.5.1 and seaborn 0.11.2 (Python). Feature importance was interpreted using SHapley Additive exPlanations (SHAP) values (shap 0.41.0, Python) with stability validated by 3-fold cross-validation. Spearman’s correlations between radiomic and pathomic features were adjusted for multiple comparisons using the Benjamini-Hochberg false discovery rate (FDR) procedure, with statistical significance defined as FDR-adjusted P (*q*-value) < 0.05.

## 3 Results

### 3.1 General information

Ninety patients diagnosed with lung adenocarcinoma were enrolled in this study, comprising 52 with LNM and 38 without LNM. The flowchart for screening patients is shown in **Supplementary Figure S1**. The cohort included 48 males and 42 females. Participants were randomly assigned to the training (*n* = 62) and testing (*n* = 28) sets in a 7:3 ratio. Statistically significant differences in CEA levels were observed between the LNM and non-LNM groups in both sets (training set: *P* = 0.004, testing set: *P* = 0.001). No statistically significant differences were noted in gender, age, smoking history, tumor location, lobulation sign, and spiculation sign between the two groups (*P* > 0.05) (**Table 1**).

**Table 1:** The clinical and radiological characteristics of patients in the training and testing sets.

Characteristics		Training set (*n* = 62)		*P*	Testing set (*n* = 28)		*P*
		LNM (*n* = 36)	Non-LNM (*n* = 26)		LNM (*n* = 16)	Non-LNM (*n* = 12)	
Gender	Male	22	12	0.243	9	5	0.704
	Female	14	14		7	7	
Age		62.03 ± 14.20	64.12 ± 13.35	0.560	67.25 ± 9.04	64.08 ± 13.67	0.467
Smoke	Yes	11	6	0.515	9	2	0.054
	No	25	20		7	10	
Location							
LUL	Yes	7	4	0.939	6	4	1.000
	No	29	22		10	8	
LLL	Yes	10	3	0.121	3	3	1.000
	No	26	23		13	9	
RUL	Yes	8	11	0.090	3	4	0.418
	No	28	15		13	8	
RML	Yes	6	1	0.243	3	0	0.238
	No	30	25		13	12	
RLL	Yes	7	7	0.487	3	1	0.613
	No	29	19		13	11	
Lobulation sign	Yes	25	15	0.340	10	6	0.702
	No	11	11		6	6	
Spiculation sign	Yes	17	17	0.156	9	7	1.000
	No	19	9		7	5	
Pleural indentation sign	Yes	23	16	0.850	14	5	0.017*
	No	13	10		2	7	
Bronchial cut-off sign	Yes	20	4	0.001*	9	5	0.704
	No	16	22		7	7	
CEA, ng/ml		14.35 (2.53, 107.60)	2.98 (2.02, 4.18)	0.004*	53.90 (16.20, 201.75)	2.97 (1.48, 5.66)	0.001*

LNM, lymph node metastasis; LUL, left upper lobe; LLL, left lower lobe; RUL, right upper lobe; RML, right middle lobe; RLL, right lower lobe; CEA, carcinoembryonic antigen; *Statistical significance (*P* < 0.05).

**Figure 2:**
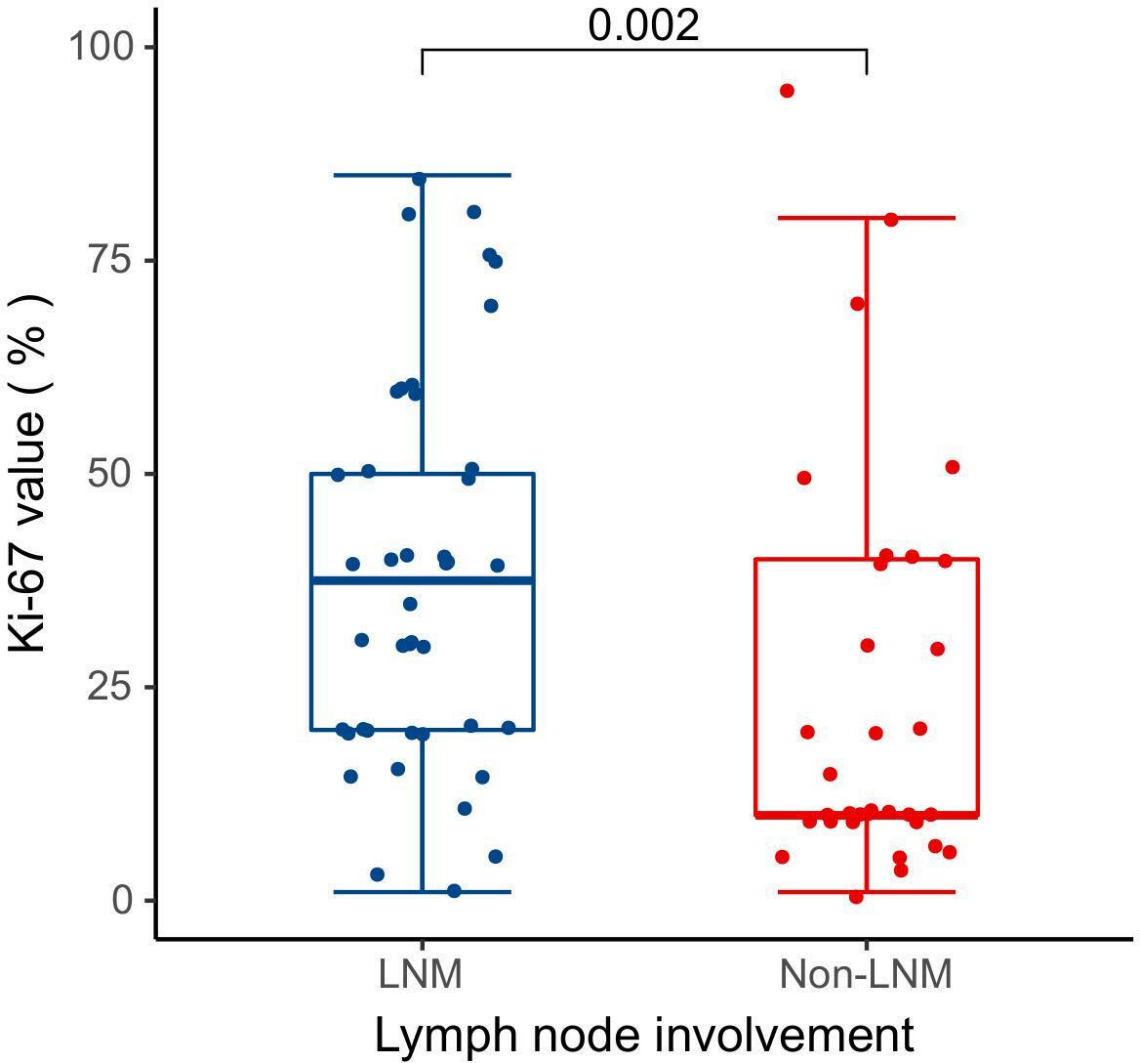
Differences in Ki-67 expression levels between the LNM and Non-LNM groups.

Pathological data were accessible for 25 patients in this study, and **Table 2** describes the pathological characteristics of the two groups. Notably, LNM in lung adenocarcinoma was significantly correlated with the presence of micropapillary component (*P* = 0.046). However, there was no statistical difference between the two groups with respect to the presence of solid component or the presence of micropapillary/solid components (*P* > 0.05). Seventy-five patients underwent immunohistochemical Ki-67 proliferation index assay, and the difference in Ki-67 expression levels between 42 LNM and 33 Non-LNM patients was statistically significant [37.50 (20.00, 50.00)% vs. 10.00 (10.00, 40.00)%, *P* = 0.002] (**Figure 2**).

**Table 2:** Differences in pathological characteristics between the LNM and Non-LNM groups.

Pathological characteristics		LNM (*n* = 5)	Non-LNM (*n* = 20)	*P*
micropapillary/solid component	Yes	5	10	0.061
	No	0	10	
micropapillary component	Yes	5	9	0.046*
	No	0	11	
solid component	Yes	1	1	0.367
	No	4	19	

*Statistical significance (*P* < 0.05).

### 3.2 Clinical risk factors

Univariate and multivariate logistic regression analyses were conducted on the clinical risk factors of patients as detailed in **Table 3**. The bronchial cut-off sign (OR = 4.55, 95%CI: 1.67-12.43, *P* = 0.003) and CEA (OR = 1.02, 95%CI: 1.00-1.04, *P* = 0.024) emerged as clinical risk factors for predicting LNM in lung adenocarcinoma.

**Table 3:** Univariate and multivariate logistic regression analysis of clinical characteristics.

Variables		Univariate analysis		Multivariate analysis	
		OR (95% CI)	*P*	OR (95% CI)	*P*
Gender		1.82 (0.78-4.25)	0.164		
Age		1.00 (0.97-1.03)	0.865		
Smoke		2.34 (0.90-6.12)	0.082		
Location	LUL	1.25 (0.46-3.40)	0.662		
	LLL	1.78 (0.61-5.21)	0.294		
	RUL	0.41 (0.16-1.04)	0.061		
	RML	7.74 (0.94-64.02)	0.058		
	RLL	0.89 (0.32-2.53)	0.831		
Lobulation sign		1.67 (0.70-3.95)	0.246		
Spiculation sign		0.58 (0.25-1.37)	0.216		
Pleural indentation sign		2.00 (0.83-4.80)	0.122		
Bronchial cut-off sign		4.06 (1.61-10.26)	0.003*	4.55 (1.67-12.43)	0.003*
CEA		1.02 (1.00-1.04)	0.019*	1.02 (1.00-1.04)	0.024*

OR, odds ratio; CI, confidence interval; LUL, left upper lobe; LLL, left lower lobe; RUL, right upper lobe; RML, right middle lobe; RLL, right lower lobe; CEA, carcinoembryonic antigen; *Statistical significance (P < 0.05).

### 3.3 Radiomic features selection

A total of 358 PET/CT radiomic features were extracted. Firstly, 156 features were eliminated by statistical methods. Then, Spearman correlation analysis was used to exclude 162 highly correlated features. Finally, 30 and 8 features were further excluded by using mRMR and GBDT algorithms, respectively. After feature selection, PET_GLRLM_LongRunHighGreyLevelEmphasis and CT_INTENSITY-BASED_IntensityBasedEnergy were retained, demonstrating a significant difference between the LNM and Non-LNM groups (*P* < 0.05) (**Table 4**). Consequently, these features were integrated to develop the combined models.

**Table 4:** Differences in selected radiomic features between the LNM and Non-LNM groups.

Radiomic features	Training set (*n* = 62)		*P*	Testing set (*n* = 28)		*P*
	LNM (*n* = 36)	Non-LNM (*n* = 26)		LNM (*n* = 16)	Non-LNM (*n* = 12)	
PET_GLRLM_LongRun HighGreyLevelEmphasis	1100.55 (784.41, 1530.53)	326.80 (176.18, 676.65)	<0.001*	955.05 (579.85, 1576.13)	333.72 (178.87, 659.85)	0.008*
CT_INTENSITY-BASED_IntensityBased Energy, ×10^8^ HU	5.05 (2.24, 7.25)	2.78 (0.85, 5.42)	0.019*	4.64 (3.07, 8.69)	1.39 (0.63, 3.06)	0.002*

*Statistical significance (*P* < 0.05).

### 3.4 Pathomic features selection

**Figure 3:**
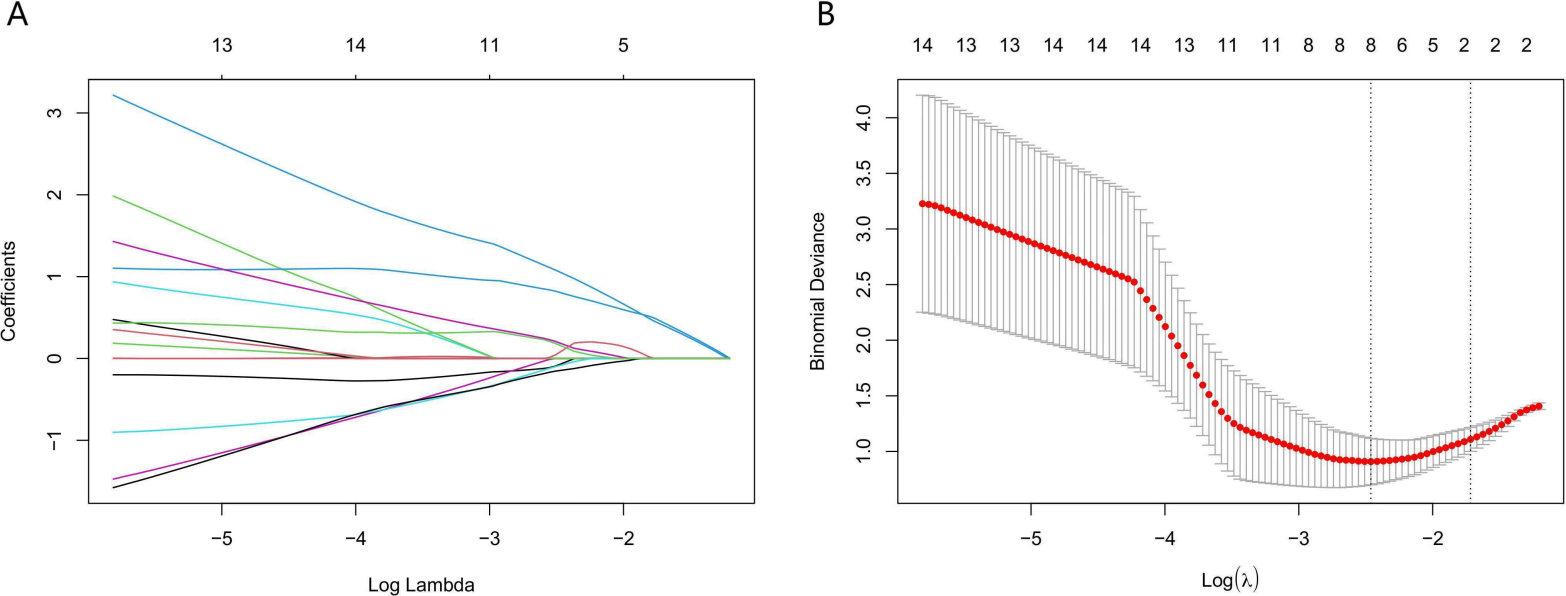
Application of the least absolute shrinkage and selection operator (LASSO) algorithm for pathomic feature selection. **(A)** LASSO coefficient curves for pathomic features. **(B)** Selection of the parameter lambda by five-fold cross-validation.

**Figure 4:**
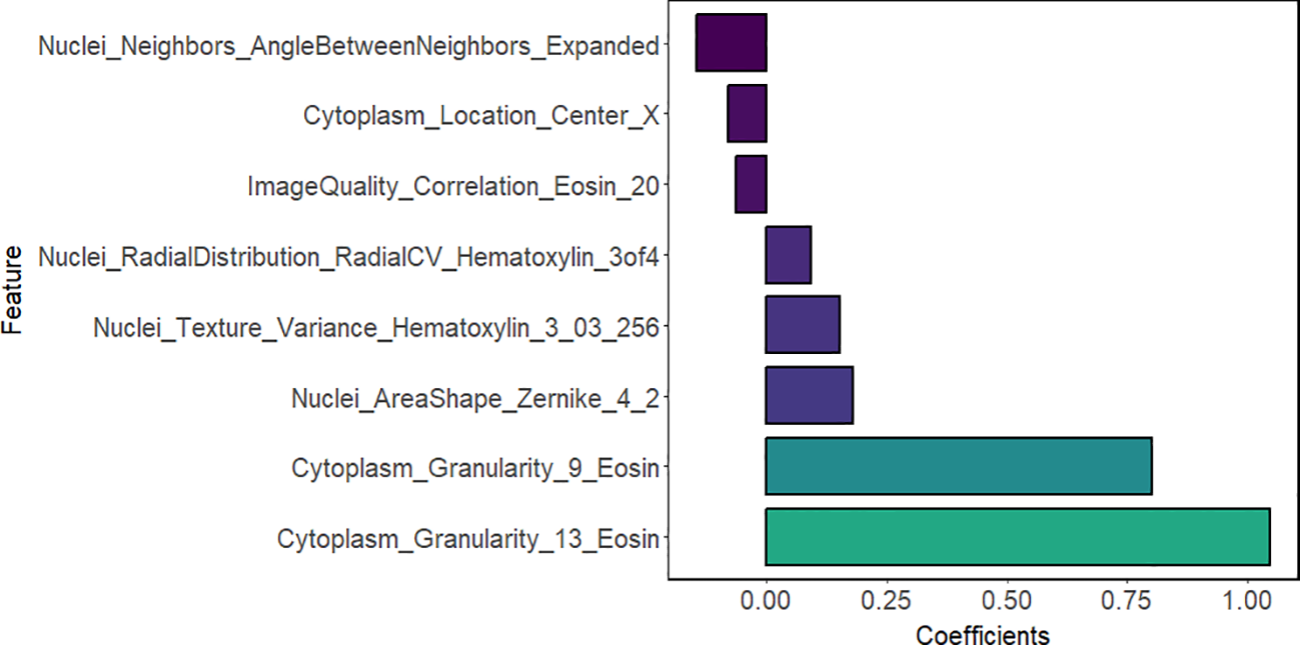
Eight selected pathomic features and their feature coefficients.

The above results indicated that LNM in lung adenocarcinoma was correlated with the presence of micropapillary component. However, differences in tumor cell morphology observed in histopathological images are not easily detected through manual inspection; instead, they could be distinguished using quantitative image features (30). Firstly, according to Spearman correlation analysis, 574 highly correlated redundant features are excluded. Then, the LASSO algorithm was used for further screening. When Lambda was 0.085 (**Figure 3**), the eight most valuable pathomic features associated with micropapillary component were retained: Nuclei_Neighbors_AngleBetweenNeighbors_Expanded, Cytoplasm_Location_Center_X, ImageQuality_Correlation_Eosin_20, Nuclei_RadialDistribution_RadialCV_Hematoxylin_3of4, Nuclei_Texture_Variance_Hematoxylin_3_03_256, Nuclei_AreaShape_Zernike_4_2, Cytoplasm_Granularity_9_Eosin, Cytoplasm_Granularity_13_Eosin (**Figure 4**).

### 3.5 Model construction and performance evaluation

**Figure 5:**
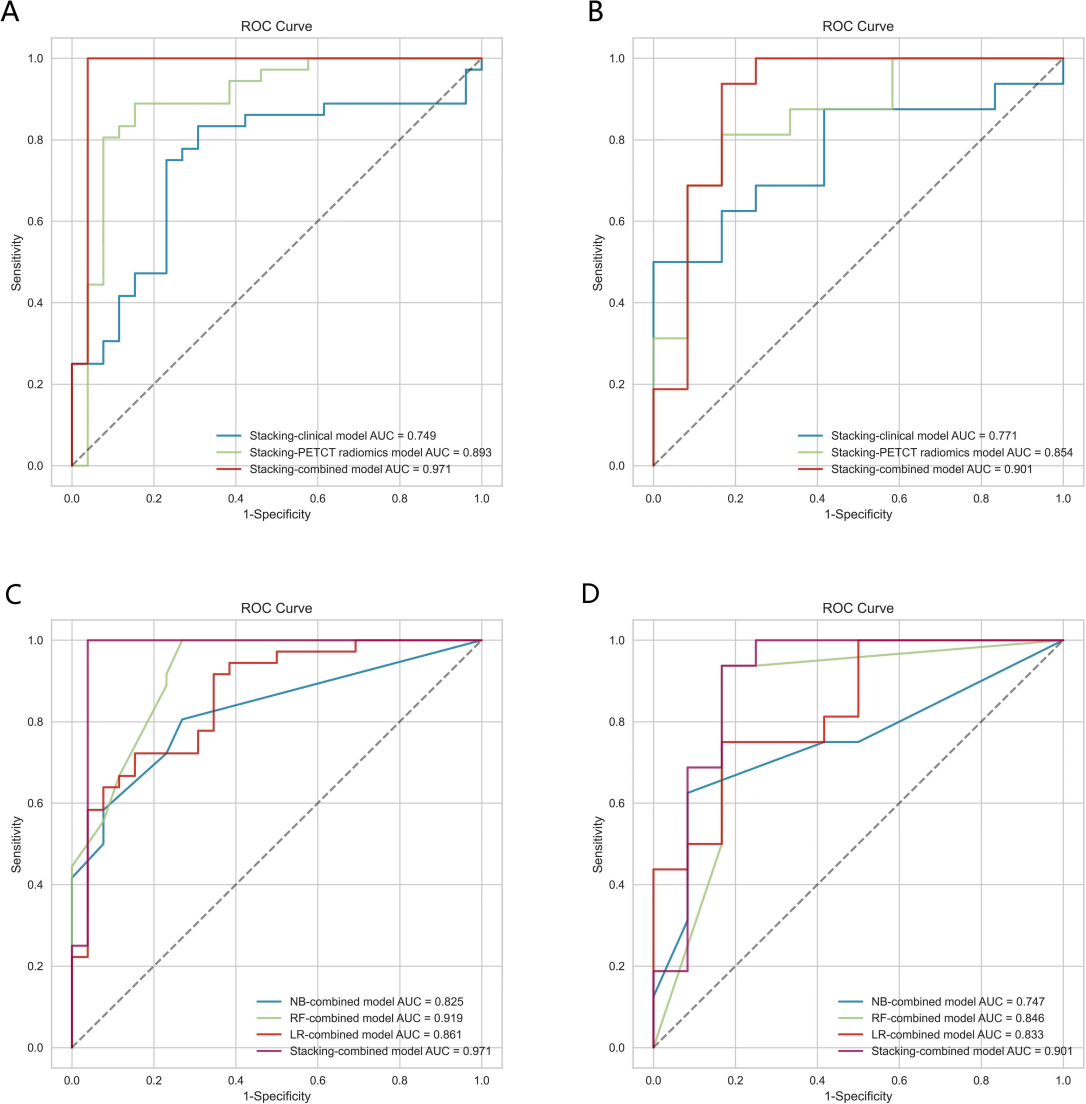
The ROC curves of models based on logistic regression, random forest, naive Bayes, and stacking ensemble learning algorithms. **(A, B)** Comparison of the stacking models in the training and testing sets. **(C, D)** Comparison of combined models in the training and testing sets.

The Receiver Operating Characteristic​ (ROC) curve analysis evaluated the clinical model, PET/CT radiomics model, and combined model utilizing the stacking ensemble learning algorithm for diagnosing LNM in lung adenocarcinoma. In the training set, the AUC values for the Stacking-clinical model, Stacking-PET/CT radiomics model, and Stacking-combined model stood at 0.749 (95% CI: 0.638-0.858), 0.893 (95% CI: 0.808-0.964), and 0.971 (95% CI: 0.917-1.000), respectively. In the testing set, the AUC values for the three models were 0.771 (95% CI: 0.615-0.914), 0.854 (95% CI: 0.719-0.959), and 0.901 (95% CI: 0.770-1.000), respectively. The Stacking-combined model demonstrated superior diagnostic efficiency, with the accuracy, sensitivity, and specificity of 0.968, 0.972, and 0.962 in the training set, and 0.857, 0.875, and 0.833 in the testing set (**Figures 5A, B**). In addition, the DeLong test for the training set indicated that the Stacking-combined model exhibited a significantly higher AUC compared to both the Stacking-PET/CT radiomics model (*P* = 0.015) and the Stacking-clinical model (*P* = 0.002). Conversely, in the testing set, the Stacking-combined model did not demonstrate a significant difference when compared to the Stacking-PET/CT radiomics model (*P* = 0.330) or the Stacking-clinical model (*P* = 0.140).

The conventional combined models utilizing LR, RF, and NB algorithms were compared to the Stacking-combined model, as depicted in **Figures 5C, D**. Analysis of the ROC curves indicated that the RF-combined model outperformed the other conventional models, with an AUC value of 0.919 (95% CI: 0.854-0.970) in the training set and an AUC value of 0.846 (95% CI: 0.706-0.971) in the testing set. The diagnostic efficacy of the Stacking-combined model for identifying LNM exceeded that of the conventional models. The DeLong test for the training set showed that the Stacking-combined model was statistically different from the LR-combined model (*P* = 0.033) and the NB-combined model (*P* = 0.003), but not significantly different from the RF-combined model (*P* = 0.210). The DeLong test for the testing set demonstrated that the Stacking-combined model was statistically different from the NB-combined model (*P* = 0.045), but not significantly different from the LR-combined model (*P* = 0.260) and the RF-combined model (*P* = 0.210). The diagnostic performance parameters in each predictive model are presented in **Table 5**.

**Table 5:** Performance parameters of each model.

Data set	Model	AUC (95% CI)	Accuracy	Sensitivity	Specificity	F1_score
Training set	Stacking-clinical model	0.749 (0.638-0.858)	0.758 (0.682-0.834)	0.806 (0.721-0.891)	0.692 (0.583-0.801)	0.795 (0.732-0.858)
	Stacking-PET/CT radiomics model	0.893 (0.808-0.964)	0.855 (0.802-0.901)	0.861 (0.803-0.907)	0.846 (0.782-0.898)	0.873 (0.821-0.915)
	Stacking-combined model	0.971 (0.917-1.000)	0.968 (0.935-0.999)	0.972 (0.945-0.999)	0.962 (0.921-0.999)	0.972 (0.951-0.993)
	LR-combined model	0.861 (0.776-0.934)	0.790 (0.715-0.865)	0.889 (0.815-0.963)	0.654 (0.547-0.761)	0.831 (0.775-0.887)
	RF-combined model	0.919 (0.854-0.970)	0.855 (0.792-0.918)	0.917 (0.853-0.981)	0.769 (0.672-0.866)	0.880 (0.832-0.928)
	NB-combined model	0.825 (0.742-0.905)	0.774 (0.701-0.847)	0.806 (0.721-0.891)	0.731 (0.632-0.830)	0.806 (0.748-0.864)
Testing set	Stacking-clinical model	0.771 (0.615-0.914)	0.714 (0.581-0.847)	0.875 (0.763-0.987)	0.500 (0.362-0.638)	0.778 (0.695-0.861)
	Stacking-PET/CT radiomics model	0.854 (0.719-0.959)	0.750 (0.683-0.809)	0.688 (0.605-0.762)	0.833 (0.752-0.897)	0.759 (0.692-0.818)
	Stacking-combined model	0.901 (0.770-1.000)	0.857 (0.768-0.946)	0.875 (0.782-0.968)	0.833 (0.715-0.951)	0.875 (0.802-0.948)
	LR-combined model	0.833 (0.695-0.947)	0.750 (0.621-0.879)	0.938 (0.882-0.994)	0.500 (0.362-0.638)	0.811 (0.723-0.900)
	RF-combined model	0.846 (0.706-0.971)	0.893 (0.815-0.971)	0.938 (0.882-0.994)	0.833 (0.715-0.951)	0.909 (0.851-0.967)
	NB-combined model	0.747 (0.584-0.892)	0.643 (0.512-0.774)	0.750 (0.615-0.885)	0.500 (0.362-0.638)	0.706 (0.615-0.797)

AUC, area under curve; CI, confidence interval; LR, logistic regression; RF, random forest; NB, naive Bayes.

### 3.6 Clinical application

**Figure 6:**
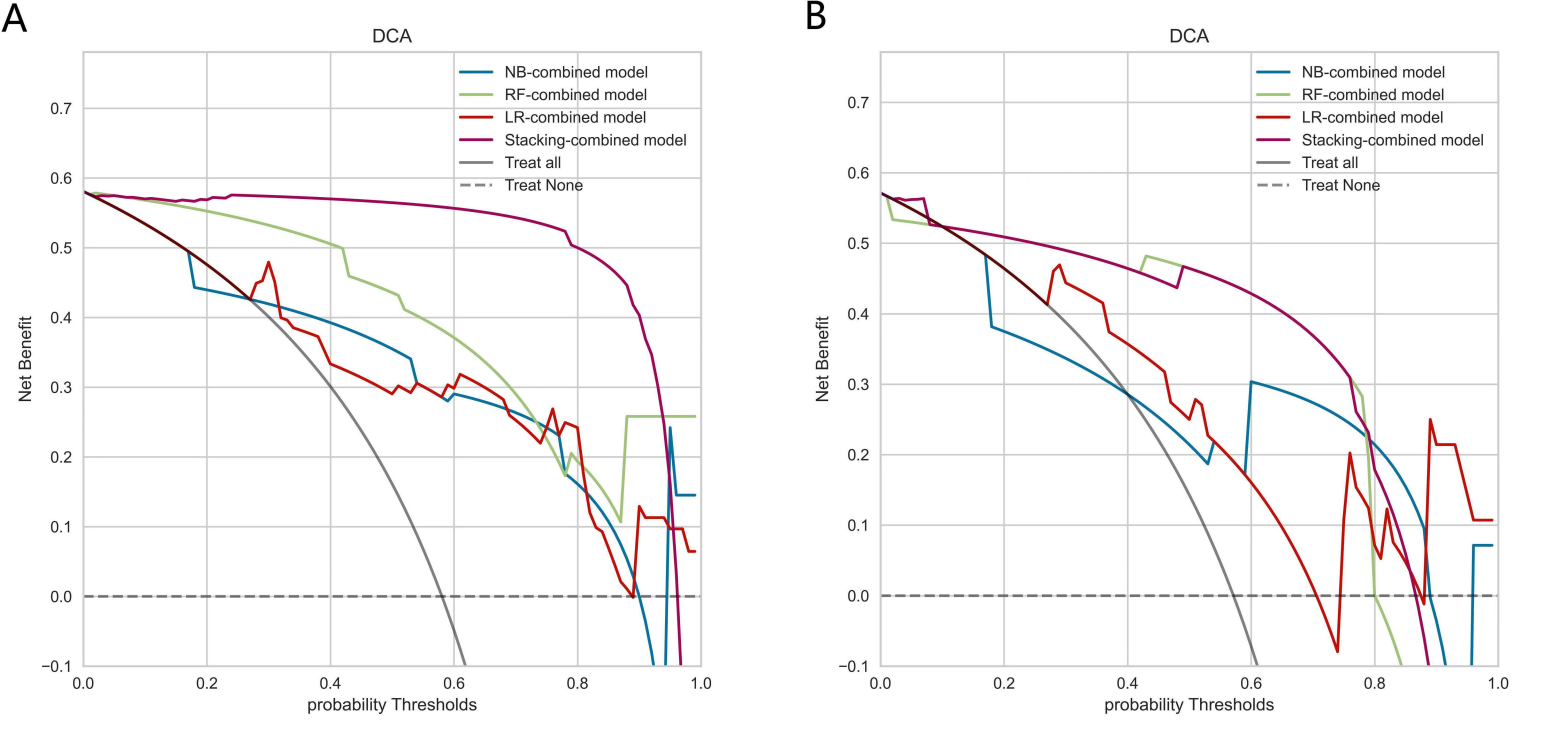
Decision curve analysis of combined models based on logistic regression, random forest, naive Bayes, and stacking ensemble learning algorithms. **(A)** Decision curves of combined models in the training set. **(B)** Decision curves of combined models in the testing set.

DCA demonstrated that the Stacking-combined model exhibits superior performance in distinguishing LNM when compared to the combined models based on LR, RF, and NB algorithms, as illustrated in **Figure 6**.

### 3.7 Radiomic features stability and contribution

Two radiomic features maintained identical ranking across all 3-fold cross-validation (**Table 6**). PET_GLRLM_LongRunHighGreyLevelEmphasis consistently ranked as the most impactful feature, with a mean SHAP value ranging from 0.30 to 0.32 (± 0.07-0.09). The second most important feature, CT_INTENSITY-BASED_IntensityBasedEnergy, showed stable performance across folds, with a mean SHAP value of 0.23 to 0.25 (± 0.05-0.07).

**Table 6:** Feature importance by 3-fold cross-validated SHAP analysis.

Cross-validation fold	Feature name	Mean SHAP value ± SD	Feature rank
Fold 1	PET_GLRLM_LongRunHighGreyLevelEmphasis	0.32 ± 0.08	1
	CT_INTENSITY-BASED_IntensityBasedEnergy	0.25 ± 0.06	2
Fold 2	PET_GLRLM_LongRunHighGreyLevelEmphasis	0.30 ± 0.07	1
	CT_INTENSITY-BASED_IntensityBasedEnergy	0.23 ± 0.05	2
Fold 3	PET_GLRLM_LongRunHighGreyLevelEmphasis	0.31 ± 0.09	1
	CT_INTENSITY-BASED_IntensityBasedEnergy	0.24 ± 0.07	2

SHAP, SHapley Additive exPlanations; SD, Standard deviation.

### 3.8 Interpretation of radiomic features

Spearman correlation analysis was utilized to evaluate the potential relationships between radiomic features and pathomic features. According to the **Table 7**, radiomic-pathomic correlations did not reach statistical significance after FDR adjustment (*q* > 0.05). However, nominal associations (uncorrected *P* < 0.05) were observed for partial feature pairs. PET_GLRLM_LongRunHighGreyLevelEmphasis demonstrated a moderate negative correlation with ImageQuality_Correlation_Eosin_20 (r = -0.422, uncorrected *P* = 0.035), as well as a moderate positive correlation with Nuclei_Texture_Variance_Hematoxylin_3_03_256 (r = 0.408, uncorrected *P* = 0.043); CT_INTENSITY-BASED_IntensityBasedEnergy demonstrated a moderate negative correlation with Cytoplasm_Location_Center_X (r = -0.407, uncorrected *P* = 0.044).

**Table 7:** Correlation analysis between radiomic features and pathomic features.

Radiomic features	Pathomic features	Correlation	*P*	*q*
PET_GLRLM_LongRunHighGreyLevelEmphasis	Nuclei_Neighbors_AngleBetweenNeighbors_Expanded	-0.192	0.359	0.574
	Cytoplasm_Location_Center_X	-0.078	0.712	0.759
	ImageQuality_Correlation_Eosin_20	-0.422	0.035	0.232
	Nuclei_RadialDistribution_RadialCV_Hematoxylin_3of4	0.225	0.279	0.561
	Nuclei_Texture_Variance_Hematoxylin_3_03_256	0.408	0.043	0.232
	Nuclei_AreaShape_Zernike_4_2	0.245	0.239	0.561
	Cytoplasm_Granularity_9_Eosin	0.108	0.606	0.701
	Cytoplasm_Granularity_13_Eosin	0.216	0.299	0.561
CT_INTENSITY-BASED_IntensityBasedEnergy	Nuclei_Neighbors_AngleBetweenNeighbors_Expanded	0.139	0.507	0.701
	Cytoplasm_Location_Center_X	-0.407	0.044	0.232
	ImageQuality_Correlation_Eosin_20	-0.209	0.315	0.561
	Nuclei_RadialDistribution_RadialCV_Hematoxylin_3of4	0.002	0.991	0.991
	Nuclei_Texture_Variance_Hematoxylin_3_03_256	0.106	0.614	0.701
	Nuclei_AreaShape_Zernike_4_2	-0.369	0.069	0.277
	Cytoplasm_Granularity_9_Eosin	0.129	0.538	0.701
	Cytoplasm_Granularity_13_Eosin	0.295	0.152	0.485

In terms of the association between radiomic features and Ki-67 expression levels, PET_GLRLM_LongRunHighGreyLevelEmphasis showed a significantly stronger positive correlation with Ki-67 expression level (r = 0.610, *q* < 0.001); CT_INTENSITY-BASED_ IntensityBasedEnergy showed a significant moderate positive correlation with Ki-67 expression level (r = 0.332, *q* = 0.004) (**Table 8**).

**Table 8:** Correlation analysis between radiomic features and Ki-67 expression levels.

Radiomic features	Variable	Correlation	*P*	*q*
PET_GLRLM_LongRunHighGreyLevelEmphasis	Ki-67 value	0.610	<0.001	<0.001*
CT_INTENSITY-BASED_IntensityBasedEnergy	Ki-67 value	0.332	0.004	0.004*

*Statistical significance (*q* < 0.05).

## 4 Discussion

LNM is an important prognostic factor for patients with lung adenocarcinoma, and accurate prediction of LNM is crucial for determining appropriate treatment strategies. In this study, we developed a stacking ensemble learning model to predict LNM through leveraging the diversity and complementarity of various machine learning models. The predictive performance of the Stacking-combined model outperformed the Stacking-clinical model and Stacking-PET/CT radiomics model, as well as LR, RF, and NB-combined models. And this study revealed a correlation between pathomic features and both PET texture feature and CT intensity feature.

By integrating clinical information of patients’ potential triggers, CT images reflecting morphology, and PET images reflecting molecular metabolism, the proposed Stacking-combined model demonstrated superior predictive performance compared to the Stacking-PET/CT radiomics model and the Stacking-clinical model in both the training and testing sets. It provides a more comprehensive approach to capture diverse characteristics of the tumor in all aspects. Multivariate logistic regression analysis identified serum CEA levels and bronchial cut-off sign as significant clinical risk factors. Previous studies have similarly reported that CEA was effective in predicting LNM in lung cancer patients (31–33). Aligning with the findings of Gao et al. (34), lung adenocarcinoma with positive LNM often exhibited bronchial cut-off sign. In addition, other malignant CT imaging features have been identified as potential risk factors (35, 36). The stacking models combined clinical and radiomic features have also shown significant value in predicting LNM of other cancers. Han et al. (37) developed a stacking-combined model to predict occult lymph node metastasis in early-stage tongue cancer, which demonstrated outstanding performance with an AUC of 0.949 (radiomics model: 0.893, clinical model: 0.728, and deep learning model: 0.798). Zhu et al. (38) constructed a longitudinal stacking model with clinical and surgical factors to further improve the accuracy of sentinel lymph node metastasis for breast cancer patients after neoadjuvant chemotherapy.

Several studies have compared the performance of different machine learning algorithms for predicting LNM in NSCLC, and the results indicated that the AUC values of the most effective models ranged from 0.85 to 0.95 (14, 15, 18, 19). In this study, the AUC values of the Stacking-combined model were 0.971 in the training set and 0.901 in the testing set, respectively, demonstrating its significant application potential for predicting LNM in lung adenocarcinoma. Stacking, a heterogeneous ensemble machine learning algorithm, improves overall predictive accuracy by leveraging the strengths of each model (39, 40). It has been shown to be particularly valuable for small datasets where single algorithms may underperform. In this study, we selected LR, RF, and NB algorithms, which exhibit significant differences in principle and superior predictive performance, as the base learners. LR was chosen for its interpretability and robustness to linear relationships; RF for handling non-linear patterns and feature interactions; and NB for its efficiency with high-dimensional data. The AUC values for the three base learners were 0.861, 0.919, and 0.825 in the training set, and 0.833, 0.846, and 0.747 in the testing set, respectively. And LR was selected as the meta-learner for its simplicity and effectiveness to linearly weight base learner outputs without overcomplicating the model, ensuring stability in small samples. Subsequently, parameter tuning was performed using five-fold cross-validation to optimize the performance of each model, ensuring that the final model was not overfitted to the training data. Ultimately, the Stacking-combined model outperformed the pre-training combined models of LR, RF, and NB, showing some improvement in both sensitivity and specificity in the training set. Consistent with previous findings (41–43), stacking models have demonstrated superior predictive performance. Islam et al. (41) found that the stacking model could detect ICU admission risk in patients with COVID-19 infection, which clearly outperformed other machine learning classifiers, with AUCs of 0.90 and 0.91 for two datasets, respectively. Lee et al. (42) proposed a stacking model to predict locoregional recurrence in breast cancer patients with the highest AUC of 0.78, while the other models exhibited AUCs ranging from 0.61 to 0.70. In the study of Bi et al. (43), the diagnostic performance of the stacking model (AUC = 0.915) was better than that of the optimal radiomics model (AUC = 0.910) in the internal validation group. Overall, the stacking model demonstrates superior prediction performance in disease diagnosis and treatment compared with single machine learning models.

In this study, PET_GLRLM_LongRunHighGreyLevelEmphasis and CT_INTENSITY-BASED_IntensityBasedEnergy were considered as potential predictors of LNM in lung adenocarcinoma. And our SHAP analysis revealed that the two features had stable importance in our model, suggesting their potential biological and clinical relevance to the prediction task. LongRunHighGreyLevelEmphasis (LRHGE) serves as an indicator of texture roughness in areas with high gray levels. Energy is employed to evaluate the distribution of pixel intensity within images. The results indicated that tumors with more complex texture distribution and higher intensity levels were at a greater risk of developing LNM. GLRLM_LRHGE in PET images has also proven to be a valuable predictive indicator in different types of cancers. Gao et al. (44) identified LRHGE as a significant predictor of synchronous metastatic disease in pancreatic ductal adenocarcinoma (PDAC), with M1 patients exhibiting significantly higher LRHGE values compared to M0 patients. In the case of NSCLC, tumor microenvironment immune types-I (TMIT-I) demonstrated higher LRHGE values and were more likely to benefit from immunotherapy (45). Another study revealed that high-grade breast cancer had higher LRHGE values (46). Similarly, energy in CT images has been confirmed to reflect tumor heterogeneity, which was related to poor prognosis (47) and poor response to treatment (48); the study conducted by Barszczyk et al. (49) also demonstrated that energy was able to effectively predict axillary lymph node metastasis in patients with breast cancer. In summary, these radiomic features not only provided important information for predicting LNM in lung adenocarcinoma, but also demonstrated broad predictive potential in other cancers.

It is challenging to accurately interpret the predictive significance of individual features. In recent years, various studies have been devoted to converting radiomic features into more interpretable formats, thereby elucidating their intrinsic properties. For instance, in NSCLC patients receiving immunotherapy, the wavelet features of CT radiomics correlated greatly with the Haralick-type features of pathomics (50); the Gabor texture features of MRI showed a strong correlation with the glandular cavity shape features of prostate cancer (51); the PET texture features employed to predict pelvic lymph node metastasis and COX-2 expression in cervical cancer demonstrated a weak correlation with the corresponding features in immunohistochemistry images (52).

However, our exploratory analysis revealed nominal associations between radiomic features and partial pathomic characteristics. PET_GLRLM_LongRunHighGreyLevelEmphasis had a moderate negative correlation with ImageQuality_Correlation_Eosin_20, indicating an inverse relationship between tumor texture complexity and pathological image correlation. This finding potentially reflected that tumors with large heterogeneity had higher cell density, aligning with prior research on gastrointestinal stromal tumors (53). PET_GLRLM_LongRunHighGreyLevelEmphasis also had a moderate positive correlation with Nuclei_Texture_Variance_Hematoxylin_3_03_256, suggesting a possible link between metabolic heterogeneity and nuclear pleomorphism. CT_INTENSITY-BASED_IntensityBasedEnergy was moderately negatively correlated with Cytoplasm_Location_Center_X, hinting alterations in the size or shape of tumor cytoplasm, aligning with prior evidence on cytoplasm texture-CT radiomic feature associations (54). A study also found that the texture features of tumor nucleus and cytoplasm were correlated with CT radiomic features (54). The absence of FDR-significant radiomic-pathomic correlations in this study likely stems from limited pathological sample size and fundamental technical scale discrepancies. Future studies require spatial multi-omics integration to bridge tumor-level imaging with cellular/molecular pathology.

Notably, we found a significant positive correlation between radiomic features and high Ki-67 expression, revealing a strong association between the proliferative activity of tumor cells and the risk of LNM. Ki-67 is a nuclear antigen closely related to the cell cycle and is present in all proliferating cells. The higher its expression level, the stronger the proliferative activity of tumor cells and the relatively higher the degree of malignancy. The results of this study showed that the Ki-67 expression level in the LNM group was significantly higher than that in the Non-LNM group, which indicates that tumor cells with high proliferative activity were more likely to develop LNM (55, 56). Therefore, these radiomic features not only reflect the proliferative activity of the tumor, but also provide an important basis for clinical prediction of LNM (57).

Pathomic-radiomic correlation builds a bridge between imaging heterogeneity and tumor aggressiveness by revealing the biological mechanisms of specific radiomic features, thereby providing a more interpretable framework for AI-driven diagnostics. The clinical translation of such models requires several steps, including validation on larger, multicenter datasets, integration with existing diagnostic workflows, and development of user friendly interfaces for clinicians. Potential challenges include the need for standardized imaging protocols, the interpretation of AI-generated results, and the integration of AI models into electronic health records. Collectively, this pathomic-radiomic correlation not only describes the biological essence of imaging phenotypes but also paves a clinically actionable pathway for AI diagnostics.

This study had some limitations. Firstly, the single-center retrospective design and limited sample size inherently restrict the statistical power and generalizability of our model. Secondly, due to individual variations in patient conditions and current technological constraints, some lymph nodes have not been pathologically biopsied. Lastly, pathomic features were obtained from surgical specimens excluding puncture samples due to tissue integrity and puncture volume, which may have introduced some bias into the correlation results. Future multi-center validation cohorts are essential to externally verify model generalizability, while integrating genomic data, including spatial transcriptomics and ctDNA analysis, could synergistically uncover molecular drivers of LNM and improve the prediction model.

## 5 Conclusions

In conclusion, the stacking ensemble learning model effectively predicted LNM in lung adenocarcinoma patients based on ^18^F-FDG PET/CT radiomic features, bronchial cut-off sign and serum CEA levels. Moreover, histopathological information provides a morphological basis for the interpretation of radiomic features, expected to aid in obtaining a more accurate diagnosis and formulating more precise and personalized treatment strategies.

## Data Availability

The raw data supporting the conclusions of this article will be made available by the authors, without undue reservation.

## Supplementary material

Supplementary Figure S1 Flowchart for screening patients.

## References

[1] Luo G Zhang Y Etxeberria J Arnold M Cai X Hao Y. Projections of lung cancer incidence by 2035 in 40 countries worldwide: population-based study. JMIR Public Health Surveill. (2023) 9:e43651. doi: 10.2196/43651, PMID: 36800235 PMC9984998

[2] Siegel RL Miller KD Fuchs HE Jemal A. Cancer statistics, 2022. CA Cancer J Clin. (2022) 72:7–33. doi: 10.3322/caac.21708, PMID: 35020204

[3] Xu J Lai J Huang X Ren Y Chen Q Li W. Survival outcomes following complete mediastinal lymphadenectomy or selective mediastinal lymphadenectomy in patients with stage I-IIIA non-small cell lung cancer: protocol for a systematic review and meta-analysis. BMJ Open. (2024) 14:e084520. doi: 10.1136/bmjopen-2024-084520, PMID: 38458808 PMC10928774

[4] Riely GJ Wood DE Ettinger DS Aisner DL Akerley W Bauman JR. Non-small cell lung cancer, version 4.2024, NCCN clinical practice guidelines in oncology. J Natl Compr Canc Netw. (2024) 22:249–74. doi: 10.6004/jnccn.2204.0023, PMID: 38754467

[5] Huang J Osarogiagbon RU Giroux DJ Nishimura KK Bille A Cardillo G. The international association for the study of lung cancer staging project for lung cancer: proposals for the revision of the N descriptors in the forthcoming ninth edition of the TNM classification for lung cancer. J Thorac Oncol. (2024) 19:766–85. doi: 10.1016/j.jtho.2023.10.012, PMID: 37866624 PMC12323887

[6] Guo W Lv B Yang T Tian M Liu M Lin X. Role of dynamic contrast-enhanced magnetic resonance imaging parameters and extracellular volume fraction as predictors of lung cancer subtypes and lymph node status in non-small-cell lung cancer patients. J Cancer. (2023) 14:3108–16. doi: 10.7150/jca.88367, PMID: 37859821 PMC10583593

[7] Owens C Hindocha S Lee R Millard T Sharma B. The lung cancers: staging and response, CT, 18F-FDG PET/CT, MRI, DWI: review and new perspectives. Br J Radiol. (2023) 96:20220339. doi: 10.1259/bjr.20220339, PMID: 37097296 PMC10392646

[8] Al-Ibraheem A Hirmas N Fanti S Paez D Abuhijla F Al-Rimawi D. Impact of 18F-FDG PET/CT, CT and EBUS/TBNA on preoperative mediastinal nodal staging of NSCLC. BMC Med Imaging. (2021) 21:49. doi: 10.1186/s12880-021-00580-w, PMID: 33731050 PMC7967993

[9] Bedetti B Schnorr P May S Ruhlmann J Ahmadzadehfar H Essler M. Multidisciplinary postoperative validation of 18F-FDG PET/CT scan in nodal staging of resected non-small cell lung cancer. J Clin Med. (2022) 11:7215. doi: 10.3390/jcm11237215, PMID: 36498790 PMC9741057

[10] Guiot J Vaidyanathan A Deprez L Zerka F Danthine D Frix AN. A review in radiomics: Making personalized medicine a reality via routine imaging. Med Res Rev. (2022) 42:426–40. doi: 10.1002/med.21846, PMID: 34309893

[11] Anan N Zainon R Tamal M. A review on advances in 18F-FDG PET/CT radiomics standardization and application in lung disease management. Insights Imaging. (2022) 13:22. doi: 10.1186/s13244-021-01153-9, PMID: 35124733 PMC8817778

[12] Nakajo M Jinguji M Ito S Tani A Hirahara M Yoshiura T. Clinical application of 18F-fluorodeoxyglucose positron emission tomography/computed tomography radiomics-based machine learning analyses in the field of oncology. Jpn J Radiol. (2024) 42:28–55. doi: 10.1007/s11604-023-01476-1, PMID: 37526865 PMC10764437

[13] Zheng K Wang X Jiang C Tang Y Fang Z Hou J. Pre-operative prediction of mediastinal node metastasis using radiomics model based on (18)F-FDG PET/CT of the primary tumor in non-small cell lung cancer patients. Front Med (Lausanne). (2021) 8:673876. doi: 10.3389/fmed.2021.673876, PMID: 34222284 PMC8249728

[14] Chang C Ruan M Lei B Yu H Zhao W Ge Y. Development of a PET/CT molecular radiomics-clinical model to predict thoracic lymph node metastasis of invasive lung adenocarcinoma ≤ 3 cm in diameter. EJNMMI Res. (2022) 12:23. doi: 10.1186/s13550-022-00895-x, PMID: 35445899 PMC9023644

[15] Dai M Wang N Zhao X Zhang J Zhang Z Zhang J. Value of presurgical (18)F-FDG PET/CT radiomics for predicting mediastinal lymph node metastasis in patients with lung adenocarcinoma. Cancer Biother Radiopharm. (2024) 39:600–10. doi: 10.1089/cbr.2022.0038., PMID: 36342812

[16] Huang Y Jiang X Xu H Zhang D Liu LN Xia YX. Preoperative prediction of mediastinal lymph node metastasis in non-small cell lung cancer based on 18F-FDG PET/CT radiomics. Clin Radiology. (2023) 78:8–17. doi: 10.1016/j.crad.2022.08.140, PMID: 36192203

[17] Qiao J Zhang X Du M Wang P Xin J. (18)F-FDG PET/CT radiomics nomogram for predicting occult lymph node metastasis of non-small cell lung cancer. Front Oncol. (2022) 12:974934. doi: 10.3389/fonc.2022.974934, PMID: 36249026 PMC9554943

[18] Yoo J Cheon M Park YJ Hyun SH Zo JI Um SW. Machine learning-based diagnostic method of pre-therapeutic 18F-FDG PET/CT for evaluating mediastinal lymph nodes in non-small cell lung cancer. Eur Radiol. (2021) 31:4184–94. doi: 10.1007/s00330-020-07523-z, PMID: 33241521

[19] Rogasch JMM Michaels L Baumgärtner GL Frost N Rückert JC Neudecker J. A machine learning tool to improve prediction of mediastinal lymph node metastases in non-small cell lung cancer using routinely obtainable [18F]FDG-PET/CT parameters. Eur J Nucl Med Mol Imaging. (2023) 50:2140–51. doi: 10.1007/s00259-023-06145-z, PMID: 36820890 PMC10199849

[20] Chang C Sun X Zhao W Wang R Qian X Lei B. Minor components of micropapillary and solid subtypes in lung invasive adenocarcinoma (≤ 3 cm): PET/CT findings and correlations with lymph node metastasis. Radiol Med. (2020) 125:257–64. doi: 10.1007/s11547-019-01112-x, PMID: 31823295

[21] Zhao Y Wang R Shen X Pan Y Cheng C Li Y. Minor components of micropapillary and solid subtypes in lung adenocarcinoma are predictors of lymph node metastasis and poor prognosis. Ann Surg Oncol. (2016) 23:2099–105. doi: 10.1245/s10434-015-5043-9, PMID: 26842488 PMC4858562

[22] Yu KH Zhang C Berry GJ Altman RB Ré C Rubin DL. Predicting non-small cell lung cancer prognosis by fully automated microscopic pathology image features. Nat Commun. (2016) 7:12474. doi: 10.1038/ncomms12474, PMID: 27527408 PMC4990706

[23] Chen D Lai J Cheng J Fu M Lin L Chen F. Predicting peritoneal recurrence in gastric cancer with serosal invasion using a pathomics nomogram. iScience. (2023) 26:106246. doi: 10.1016/j.isci.2023.106246, PMID: 36994190 PMC10040964

[24] Gilley P Zhang K Abdoli N Sadri Y Adhikari L Fung KM. Utilizing a pathomics biomarker to predict the effectiveness of bevacizumab in ovarian cancer treatment. Bioengineering (Basel). (2024) 11:678. doi: 10.3390/bioengineering11070678, PMID: 39061760 PMC11273783

[25] Alvarez-Jimenez C Sandino AA Prasanna P Gupta A Viswanath SE Romero E. Identifying cross-scale associations between radiomic and pathomic signatures of non-small cell lung cancer subtypes: preliminary results. Cancers (Basel). (2020) 12:3663. doi: 10.3390/cancers12123663, PMID: 33297357 PMC7762258

[26] Shiradkar R Panda A Leo P Janowczyk A Farre X Janaki N. T1 and T2 MR fingerprinting measurements of prostate cancer and prostatitis correlate with deep learning-derived estimates of epithelium, lumen, and stromal composition on corresponding whole mount histopathology. Eur Radiol. (2021) 31:1336–46. doi: 10.1007/s00330-020-07214-9, PMID: 32876839 PMC7882016

[27] Brancato V Cavaliere C Garbino N Isgrò F Salvatore M Aiello M. The relationship between radiomics and pathomics in Glioblastoma patients: Preliminary results from a cross-scale association study. Front Oncol. (2022) 12:1005805. doi: 10.3389/fonc.2022.1005805, PMID: 36276163 PMC9582951

[28] Wang L Li T Hong J Zhang M Ouyang M Zheng X. 18F-FDG PET-based radiomics model for predicting occult lymph node metastasis in clinical N0 solid lung adenocarcinoma. Quant Imaging Med Surg. (2021) 11:215–25. doi: 10.21037/qims-20-337, PMID: 33392023 PMC7719913

[29] Vahadane A Peng T Sethi A Albarqouni S Wang L Baust M. Structure-preserving color normalization and sparse stain separation for histological images. IEEE Trans Med Imaging. (2016) 35:1962–71. doi: 10.1109/TMI.2016.2529665, PMID: 27164577

[30] Carpenter AE Jones TR Lamprecht MR Clarke C Kang IH Friman O. CellProfiler: image analysis software for identifying and quantifying cell phenotypes. Genome Biol. (2006) 7:R100. doi: 10.1186/gb-2006-7-10-r100, PMID: 17076895 PMC1794559

[31] Chen P Rojas FR Hu X Serrano A Zhu B Chen H. Pathomic features reveal immune and molecular evolution from lung preneoplasia to invasive adenocarcinoma. Mod Pathol. (2023) 36:100326. doi: 10.1016/j.modpat.2023.100326, PMID: 37678674 PMC10841057

[32] Liao X Liu M Li S Huang W Guo C Liu J. The value on SUV-derived parameters assessed on 18F-FDG PET/CT for predicting mediastinal lymph node metastasis in non-small cell lung cancer. BMC Med Imaging. (2023) 23:49. doi: 10.1186/s12880-023-01004-7, PMID: 37020286 PMC10077668

[33] Miao H Shaolei L Nan L Yumei L Shanyuan Z Fangliang L. Occult mediastinal lymph node metastasis in FDG-PET/CT node-negative lung adenocarcinoma patients: Risk factors and histopathological study. Thorac Cancer. (2019) 10:1453–60. doi: 10.1111/1759-7714.13093, PMID: 31127706 PMC6558456

[34] Gao Z Wang X Zuo T Zhang M Zhang Z. A predictive nomogram for lymph node metastasis in part-solid invasive lung adenocarcinoma: A complement to the IASLC novel grading system. Front Oncol. (2022) 12:916889. doi: 10.3389/fonc.2022.916889, PMID: 36046052 PMC9423719

[35] Zhang W Mu G Huang J Bian C Wang H Gu Y. Lymph node metastasis and its risk factors in T1 lung adenocarcinoma. Thorac Cancer. (2023) 14:2993–3000. doi: 10.1111/1759-7714.15088, PMID: 37667435 PMC10599970

[36] Ke L Ma H Zhang Q Wang Y Xia P Yu L. The pattern of lymph node metastasis in peripheral pulmonary nodules patients and risk prediction models. Front Surg. (2022) 9:981313. doi: 10.3389/fsurg.2022.981313, PMID: 36017514 PMC9395917

[37] Han W Wang Y Li T Dong Y Dang Y He L. A CT-based integrated model for preoperative prediction of occult lymph node metastasis in early tongue cancer. PeerJ. (2024) 12:e17254. doi: 10.7717/peerj.17254, PMID: 38685941 PMC11057426

[38] Zhu T Huang YH Li W Zhang YM Lin YY Cheng MY. Multifactor artificial intelligence model assists axillary lymph node surgery in breast cancer after neoadjuvant chemotherapy: multicenter retrospective cohort study. Int J Surg. (2023) 109:3383–94. doi: 10.1097/JS9.0000000000000621, PMID: 37830943 PMC10651262

[39] Naimi AI Balzer LB. Stacked generalization: an introduction to super learning. Eur J Epidemiol. (2018) 33:459–64. doi: 10.1007/s10654-018-0390-z, PMID: 29637384 PMC6089257

[40] Mahajan P Uddin S Hajati F Moni MA. Ensemble learning for disease prediction: A review. Healthcare (Basel). (2023) 11:1808. doi: 10.3390/healthcare11121808, PMID: 37372925 PMC10298658

[41] Islam KR Kumar J Tan TL Reaz MBI Rahman T Khandakar A. Prognostic model of ICU admission risk in patients with COVID-19 infection using machine learning. Diagnostics (Basel). (2022) 12:2144. doi: 10.3390/diagnostics12092144, PMID: 36140545 PMC9498213

[42] Lee J Yoo SK Kim K Lee BM Park VY Kim JS. Machine learning−based radiomics models for prediction of locoregional recurrence in patients with breast cancer. Oncol Lett. (2023) 26:422. doi: 10.3892/ol.2023.14008, PMID: 37664669 PMC10472028

[43] Bi Q Wang Y Deng Y Liu Y Pan Y Song Y. Different multiparametric MRI-based radiomics models for differentiating stage IA endometrial cancer from benign endometrial lesions: A multicenter study. Front Oncol. (2022) 12:939930. doi: 10.3389/fonc.2022.939930, PMID: 35992858 PMC9389365

[44] Gao J Huang X Meng H Zhang M Zhang X Lin X. Performance of multiparametric functional imaging and texture analysis in predicting synchronous metastatic disease in pancreatic ductal adenocarcinoma patients by hybrid PET/MR: initial experience. Front Oncol. (2020) 10:198. doi: 10.3389/fonc.2020.00198, PMID: 32158690 PMC7052324

[45] Zhou J Zou S Kuang D Yan J Zhao J Zhu X. A novel approach using FDG-PET/CT-based radiomics to assess tumor immune phenotypes in patients with non-small cell lung cancer. Front Oncol. (2021) 11:769272. doi: 10.3389/fonc.2021.769272, PMID: 34868999 PMC8635743

[46] Acar E Turgut B Yiğit S Kaya G. Comparison of the volumetric and radiomics findings of 18F-FDG PET/CT images with immunohistochemical prognostic factors in local/locally advanced breast cancer. Nucl Med Commun. (2019) 40:764–72. doi: 10.1097/MNM.0000000000001019, PMID: 30925542

[47] Kim C Cho HH Choi JY Franks TJ Han J Choi Y. Pleomorphic carcinoma of the lung: Prognostic models of semantic, radiomics and combined features from CT and PET/CT in 85 patients. Eur J Radiol Open. (2021) 8:100351. doi: 10.1016/j.ejro.2021.100351, PMID: 34041307 PMC8141891

[48] Kinsey CM San José Estépar R Bates JHT Cole BF Washko G Jantz M. Tumor density is associated with response to endobronchial ultrasound-guided transbronchial needle injection of cisplatin. J Thorac Dis. (2020) 12:4825–32. doi: 10.21037/jtd-20-674, PMID: 33145055 PMC7578514

[49] Barszczyk M Singh N Alikhassi A Van Oirschot M Kuling G Kiss A. 3D CT radiomic analysis improves detection of axillary lymph node metastases compared to conventional features in patients with locally advanced breast cancer. J Breast Imaging. (2024) 6:397–406. doi: 10.1093/jbi/wbae022, PMID: 38752527

[50] Dia AK Ebrahimpour L Yolchuyeva S Tonneau M Lamaze FC Orain M. The cross-scale association between pathomics and radiomics features in immunotherapy-treated NSCLC patients: A preliminary study. Cancers (Basel). (2024) 16:348. doi: 10.3390/cancers16020348, PMID: 38254838 PMC10813866

[51] Penzias G Singanamalli A Elliott R Gollamudi J Shih N Feldman M. Identifying the morphologic basis for radiomic features in distinguishing different Gleason grades of prostate cancer on MRI: Preliminary findings. PloS One. (2018) 13:e0200730. doi: 10.1371/journal.pone.0200730, PMID: 30169514 PMC6118356

[52] Zhang Z Li X Sun H. Development of machine learning models integrating PET/CT radiomic and immunohistochemical pathomic features for treatment strategy choice of cervical cancer with negative pelvic lymph node by mediating COX-2 expression. Front Physiol. (2022) 13:994304. doi: 10.3389/fphys.2022.994304, PMID: 36311222 PMC9614332

[53] Song H Xiao X Han X Sun Y Zheng G Miao Q. Development and interpretation of a multimodal predictive model for prognosis of gastrointestinal stromal tumor. NPJ Precis Oncol. (2024) 8:157. doi: 10.1038/s41698-024-00636-4, PMID: 39060449 PMC11282065

[54] Wu P Wu K Li Z Liu H Yang K Zhou R. Multimodal investigation of bladder cancer data based on computed tomography, whole slide imaging, and transcriptomics. Quant Imaging Med Surg. (2023) 13:1023–35. doi: 10.21037/qims-22-679, PMID: 36819263 PMC9929396

[55] Li Z Li F Pan C He Z Pan X Zhu Q. Tumor cell proliferation (Ki-67) expression and its prognostic significance in histological subtypes of lung adenocarcinoma. Lung Cancer. (2021) 154:69–75. doi: 10.1016/j.lungcan.2021.02.009, PMID: 33626488

[56] Hwang I Song JS Cho E Song KH Ra SH Choi CM. PPIB/Cyclophilin B expression associates with tumor progression and unfavorable survival in patients with pulmonary adenocarcinoma. Am J Cancer Res. (2024) 14:917–30. doi: 10.62347/TYNU2341, PMID: 38455410 PMC10915315

[57] Bicci E Cozzi D Cavigli E Ruzga R Bertelli E Danti G. Reproducibility of CT radiomic features in lung neuroendocrine tumors (NETs) patients: analysis in a heterogeneous population. Radiol Med. (2023) 128:203–11. doi: 10.1007/s11547-023-01592-y, PMID: 36637739 PMC9938819

