# Correction: Prediction of lymph node metastasis in lung adenocarcinoma using a PET/CT radiomics-based ensemble learning model and its pathological basis

**DOI:** 10.3389/fonc.2025.1704648

**Published:** 2025-10-02

**Authors:** Shulin Li, Fang Chen, Lei Wang, Zhiming Xiang

**Affiliations:** ^1^ Postgraduate Cultivation Base of Guangzhou University of Chinese Medicine, Panyu Central Hospital, Guangzhou, China; ^2^ Department of Radiology, The Affiliated Panyu Central Hospital, Guangzhou Medical University, Guangzhou, China; ^3^ Department of Pathology, The Affiliated Panyu Central Hospital, Guangzhou Medical University, Guangzhou, China

**Keywords:** lung adenocarcinoma, lymph node metastasis, positron emission tomography, radiomics, pathomics, stacking ensemble learning

“Nioche C, Orlhac F, Boughdad S, ReuzéS, Goya-Outi J, Robert C, et al. LIFEx: A Freeware for Radiomic Feature Calculation in Multimodality Imaging to Accelerate Advances in the Characterization of Tumor Heterogeneity. Cancer Res (2018) 78:4786–4789. doi: 10.1158/0008-5472.CAN-18-0125” was not cited in the article. The citation has now been inserted in the section **2 Materials and methods**, *2.4 PET/CT image processing and feature extraction*, Paragraph 1 and should read: “In this study, the lesion’s ROI delineation and feature extraction of PET/CT images were completed on LIFEx 7.4.0 (28).”

“Bankhead P, Loughrey MB, Fernández JA, Dombrowski Y, McArt DG, Dunne PD, et al. QuPath: Open source software for digital pathology image analysis. Sci Rep (2017) 7:16878. doi: 10.1038/s41598-017-17204-5” was not cited in the article. The citation has now been inserted in the section **2 Materials and methods**, *2.7 WSI processing and feature extraction*, Paragraph 1 and should read: “Tumor regions within WSIs were manually delineated utilizing Qupath 0.5.1 (29) and the feature extraction were completed on CellProfiler 4.2.7 (30).”

Reference 28 (Wang et al., 2021) and reference 32 (Liao et al., 2023) have been removed from the reference list.

Reference 31 was erroneously written as “Chen P, Rojas FR, Hu X, Serrano A, Zhu B, Chen H, et al. Pathomic features reveal immune and molecular evolution from lung preneoplasia to invasive adenocarcinoma. Mod Pathol. (2023) 36:100326. doi: 10.1016/j.modpat.2023.100326. It should be corrected to the original Reference 29: “Vahadane A, Peng T, Sethi A, Albarqouni S, Wang L, Baust M, et al. Structure-preserving color normalization and sparse stain separation for histological images. IEEE Trans Med Imaging. (2016) 35:1962–71. doi: 10.1109/TMI.2016.2529665.”

Reference 32 was erroneously written as “Liao X, Liu M, Li S, Huang W, Guo C, Liu J, et al. The value on SUV-derived parameters assessed on 18F-FDG PET/CT for predicting mediastinal lymph node metastasis in non-small cell lung cancer. BMC Med Imaging. (2023) 23:49. doi: 10.1186/s12880-023-01004-7”. It should be corrected to the original Reference 31: “Chen P, Rojas FR, Hu X, Serrano A, Zhu B, Chen H, et al. Pathomic features reveal immune and molecular evolution from lung preneoplasia to invasive adenocarcinoma. Mod Pathol. (2023) 36:100326. doi: 10.1016/j.modpat.2023.100326.”

A correction has been made to the section **2 Materials and methods**, *2.4 PET/CT image processing and feature extraction*, Paragraph 1:

“In this study, the lesion’s ROI delineation and feature extraction of PET/CT images were completed on LIFEx 7.4.0 (28).”

A correction has been made to the section **2 Materials and methods**, *2.7 WSI processing and feature extraction*, Paragraph 1:

“Tumor regions within WSIs were manually delineated utilizing Qupath 0.5.1 (29) and the feature extraction were completed on CellProfiler 4.2.7 (30).”

“All patches underwent color normalization utilizing the Vahadane method (31) for subsequent processing.”

A correction has been made to the section **3 Results**, *3.4 Pathomic features selection*, Paragraph 1:

“However, differences in tumor cell morphology observed in histopathological images are not easily detected through manual inspection; instead, they could be distinguished using quantitative image features (32).”

A correction has been made to the section **4 Discussion**, Paragraph 2:

“Previous studies have similarly reported that CEA was effective in predicting LNM in lung cancer patients (33).”

The original version of this article has been updated.

