# Supplementary figures and images for: Prediction of lymph node metastasis in lung adenocarcinoma using a PET/CT radiomics-based ensemble learning model and its pathological basis
Source: Front Oncol. 2025 Aug 25;15:1618494. doi: 10.3389/fonc.2025.1618494 (PMC12415029; doi:10.3389/fonc.2025.1618494)

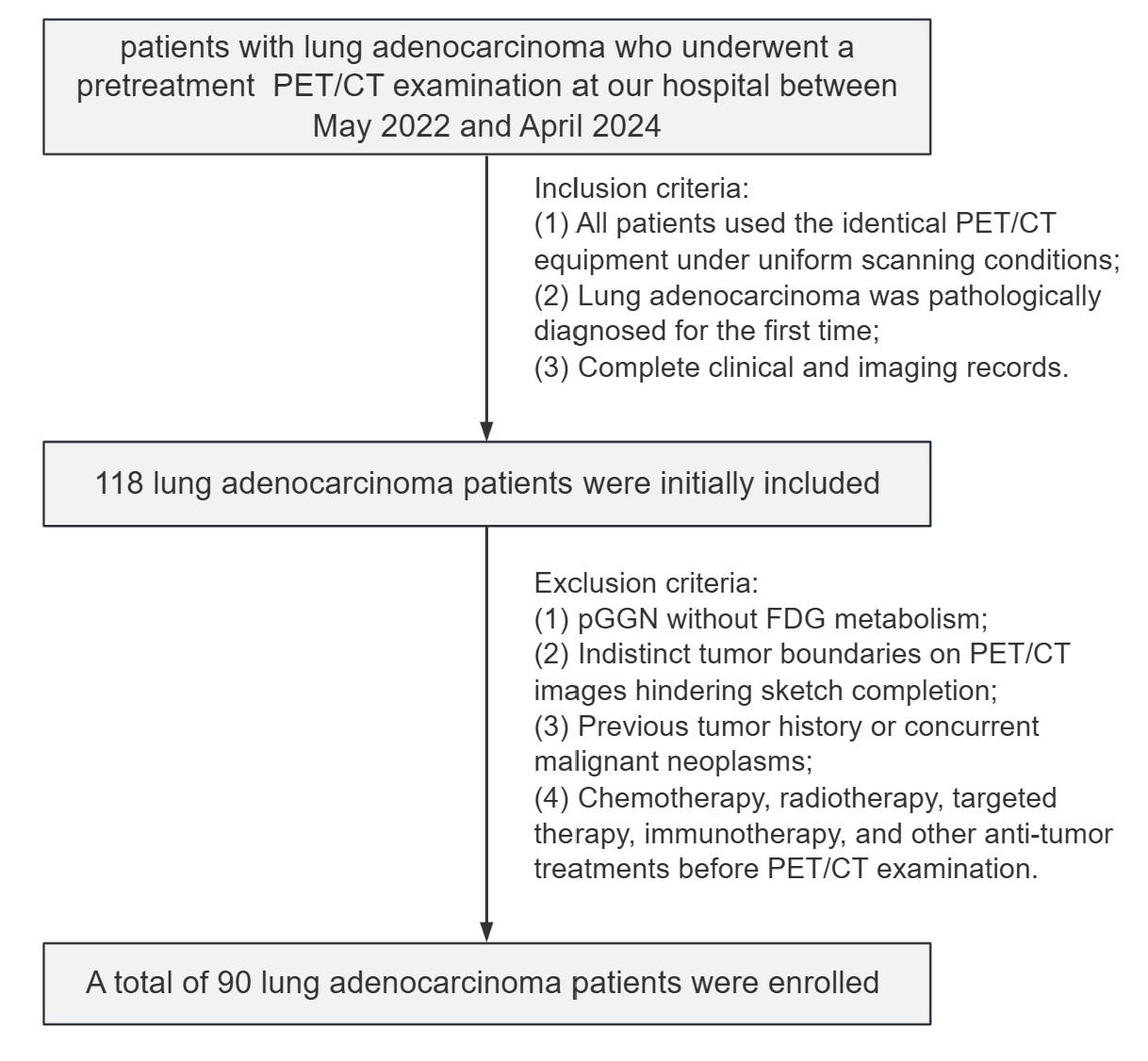

Supplement: Supplementary Figure S1 — Flowchart for screening patients. [file Image1.tif]
